# Targeting MDMX for Cancer Therapy: Rationale, Strategies, and Challenges

**DOI:** 10.3389/fonc.2020.01389

**Published:** 2020-08-05

**Authors:** De-Hua Yu, Zhi-Yuan Xu, Shaowei Mo, Li Yuan, Xiang-Dong Cheng, Jiang-Jiang Qin

**Affiliations:** ^1^College of Pharmaceutical Sciences, Zhejiang Chinese Medical University, Hangzhou, China; ^2^Institute of Cancer and Basic Medicine, Chinese Academy of Sciences, Cancer Hospital of the University of Chinese Academy of Sciences, Zhejiang Cancer Hospital, Hangzhou, China; ^3^First Clinical Medical College, Zhejiang Chinese Medical University, Hangzhou, China

**Keywords:** MDMX, MDM2, p53, oncogene, tumor suppressor, inhibitors

## Abstract

The oncogene MDMX, also known as MDM4 is a critical negative regulator of the tumor suppressor p53 and has been implicated in the initiation and progression of human cancers. Increasing evidence indicates that MDMX is often amplified and highly expressed in human cancers, promotes cancer cell growth, and inhibits apoptosis by dampening p53-mediated transcription of its target genes. Inhibiting MDMX-p53 interaction has been found to be effective for restoring the tumor suppressor activity of p53. Therefore, MDMX is becoming one of the most promising molecular targets for developing anticancer therapeutics. In the present review, we mainly focus on the current MDMX-targeting strategies and known MDMX inhibitors, as well as their mechanisms of action and *in vitro* and *in vivo* anticancer activities. We also propose other potential targeting strategies for developing more specific and effective MDMX inhibitors for cancer therapy.

## Introduction

In 1996, Shvarts et al. discovered a new binding protein of the tumor suppressor p53, which is highly homologous to a critical negative regulator of p53, i.e., the oncoprotein MDM2 (murine double minute 2) (1). Therefore, this new p53-associated protein was named MDMX (murine double minute X, also called MDM4) (1, 2). Unsurprisingly, MDMX shows functional similarity to MDM2, which binds to p53 and inhibits its transcriptional activity (3, 4). MDM2 has been identified as an E3 ubiquitin ligase to degrade p53 (5–7). Different from MDM2, MDMX does not have the E3 ligase activity and cannot directly promote the ubiquitination and degradation of p53 (8). Although it has been established that MDMX inhibits p53-mediated transactivation, regardless of MDM2 status, MDMX had been considered as a “backup” regulator of p53 for a while (9). Due to the findings that MDMX is essential for controlling p53 activity during embryonic development *in vivo* (10–12), as well as that MDMX directly interacts with MDM2 and plays an important role in enhancing MDM2's activity and stabilizing MDM2 protein (13, 14), MDMX started to be recognized as a key player in the p53-MDM2/MDMX circuitry (15–18).

The p53-MDM2 pathway has been extensively investigated during the past three decades and has been found to play critical roles in cancer initiation and progression (19–21). Because the dual functions of MDMX in the regulation of MDM2 and p53 are not redundant but essential in cancer cells, much more attention has been paid to investigate the roles of MDMX in human cancer. Recent studies have indicated that MDMX is frequently amplified and overexpressed in various types of human cancer and contributes to the development and progression of this disease. Therefore, MDMX has been demonstrated as a promising but underutilized molecular target for cancer therapy. Over the past few years, there have been several review articles on the roles of MDMX in cancer and development (15–18, 22–24), MDMX isoforms (25), inhibitors of p53-MDM2/MDMX interactions (26–29), as well as mouse modeling of MDMX (12). In the present review, we will discuss the MDMX protein structure and the role of MDMX in the p53-MDM2/MDMX loop but mainly focus on the clinical relevance of MDMX in cancer, current MDMX-targeting strategies and related MDMX inhibitors, and other potential strategies for developing new MDMX inhibitors.

## The Structure of MDMX Protein

**Figure 1:**
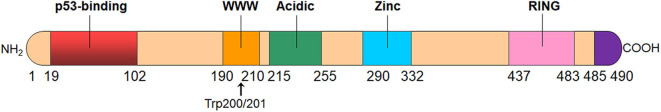
The structure of MDMX protein. MDMX has four conserved domains, including an N-terminal p53-binding domain, the central acidic domain, the zinc-finger domain, and the C-terminal RING domain. MDMX also has an autoinhibitory sequence element, i.e., WWW motif, which inhibits the MDMX-p53 interaction. The C-terminal tail (residues 485–490) of MDMX is also important for the formation of MDM2-MDMX heterodimer.

The MDMX protein consists of 490 amino acids with four main conserved domains, including the N-terminal p53-binding domain, the central acidic domain, the zinc-finger domain, and the C-terminal RING domain (Figure 1) (15, 22). The inhibitory effect of MDMX on p53-activated transcription depends on the p53-binding domain, which is strictly conserved on both MDM2 and MDMX proteins (30, 31). MDMX directly binds to the N-terminal transactivation domains (TAD) of p53 through the p53-binding domain, thereby inhibiting the transcriptional activity of p53. It has been found that MDM2 works collaboratively with MDMX to repress the transcriptional activity of p53 (32). Okamoto et al. have demonstrated that serine 367 on MDMX is critical for its binding to 14-3-3 as well as for the cooperative repression on p53 activation by MDM2 and MDMX (33). Alanine substitution at serine 367 not only blocks the binding of MDMX to 14-3-3 but also enhances cooperative inhibition of p53's transcriptional activity by MDM2 and MDMX (33). Importantly, the precise amino acids that are essential for the interactions of the p53 TAD with both MDM2 and MDMX are identical (30), leading to the development of dual inhibitors of p53-MDM2/MDMX interactions (26–29). However, the MDM2- and MDMX-binding pockets for p53 are not the same, despite the high resemblance between them (31), which results in the differences in the binding selectivity of small-molecule inhibitors to MDM2 and MDMX (34).

MDMX protein also contains a central acidic domain as MDM2. It has previously been reported that MDM2 inhibits the interaction between p53 and DNA via the acidic domain whereas MDMX only slightly mitigates this interaction (35). However, a recent study by Huang et al. has revealed that the MDMX acidic domain inhibits the p53-DNA binding and complements the role of MDM2 in controlling p53 level *in vivo* (36). Besides, the MDMX acidic domain also plays a role in the intramolecular interactions with the p53-binding domain and disrupts the p53-MDMX interaction (37, 38). The zinc-finger domain is also conserved in MDMX protein and has been found to interact with retinoblastoma protein (Rb); however, its function is yet to be determined (39). Besides, there is an MDMX autoinhibitory sequence motif, i.e., the WWW element located in a tryptophan-rich segment and centered around residues Trp200 and Trp201 in MDMX (40). This conserved WWW motif binds to MDMX's N-terminal domain and prevents its interaction with p53, while MDMX without this autoinhibitory element binds 32-fold more tightly to p53 than the full-length MDMX (40).

The C-terminal RING domain of MDMX has a 53.2% sequence identity with that of MDM2 and this RING domain is critical for the dimerization of MDM2 and MDMX proteins (41, 42). Importantly, seven amino acid residues (485–491) at the extreme C terminus plays a crucial role in the interactions among the RING domains (43, 44). The MDM2-MDMX heterodimerization is much more stable than the homodimerization of either protein (41). The RING domain of MDM2 has sufficient E3 ubiquitin ligase activity, whereas MDMX does not have the intrinsic E3 ligase function (42, 45). Although MDMX does not directly promote the ubiquitination and degradation of p53, this protein also influences the levels of both p53 and MDM2 by interacting with MDM2 (13). The C-terminal tail of MDMX can substitute for that of MDM2 and reactivate the E3 ligase function of MDM2 protein with mutated C-terminal domain, suggesting that MDMX can actively modulate MDM2's E3 activity and the C-terminal tail of MDMX plays an essential role in the cooperativity of MDMX in MDM2 ubiquitin ligase activity (43, 44). It has been found that the RING domain-mediated interaction between MDM2 and MDMX not only increases the steady-state level of MDM2 but also enhances its E3 ligase activity (13, 46). Indeed, the MDM2-MDMX hetero-RING complexes have a greater E3 ligase activity than the MDM2 homo-RING complexes, which emphasizes the importance of the MDM2-MDMX heterodimer in p53 control (46).

## The Role of MDMX in p53-MDM2/MDMX Loop

**Figure 2:**
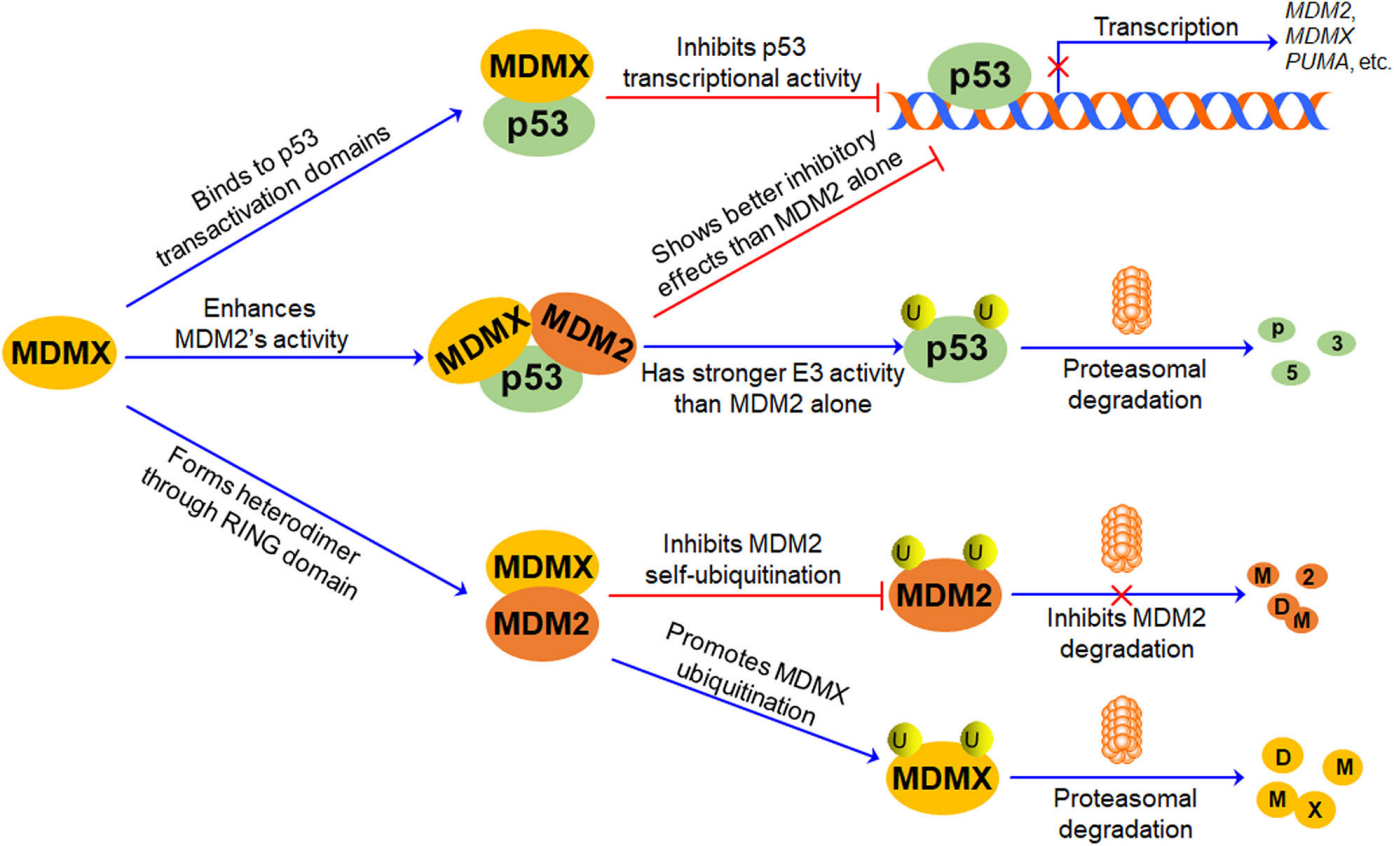
The role of MDMX in p53-MDM2/MDMX Loop. MDMX directly binds to p53 transactivation domains through its N-terminal p53-binding domain and inhibits p53-mediated transcription. MDMX also interacts with MDM2 via their RING domains and forms MDM2-MDMX heterodimer, increasing MDM2 protein stability and enhancing MDM2-mediated MDMX protein ubiquitination and degradation. Besides, the MDM2-MDMX complex shows better inhibitory effects on p53 than MDM2 alone, repressing p53-mediated transcription and promoting p53 ubiquitination and proteasomal degradation.

The p53-MDM2 feedback loop has been comprehensively discussed in previous review articles (9, 22, 47, 48). In brief, MDM2 is a transcriptional target and a negative regulator of p53 (Figure 2). MDM2 directly binds to p53 TAD and inhibits p53-mediated transcription (49, 50); it also promotes p53 polyubiquitination and proteasomal degradation (5–7). Conversely, p53 specifically binds to the *MDM2* P2 promoter and activates its transcription, thus forming an autoregulatory feedback loop (51).

Accumulating evidence suggests that MDMX plays a pivotal role in the regulation of MDM2 and p53, thereby forming the p53-MDM2/MDMX loop (Figure 2). As mentioned above, MDMX inactivates p53 through interacting with p53 and repressing its transcriptional activity, but it does not directly affect p53 protein stability (1, 2, 52). The MDMX phosphorylation by casein kinase 1α (CK1α) is required for the MDMX-p53 interaction as well as MDMX-mediated p53 inactivation (53). Besides, MDMX is a p53 target gene with a weaker p53-inducible P2 promoter (54). Furthermore, MDMX interacts with MDM2 through their respective RING domains and forms heterodimers, thereby enhancing E3 ubiquitin ligase activity of MDM2 and promoting MDM2-mediated p53 ubiquitination (46). However, upon DNA damage, MDMX interacts with p53 and induces a conformational change of p53 protein toward an active form, protecting p53 from proteasomal degradation in an MDM2- and ubiquitin-independent manner, whereas MDM2 lacks this activity (55). Interestingly, the formation of the MDM2-MDMX complex not only increases the stability of MDM2 but also causes the degradation of MDMX (13). The MDM2-MDMX heterodimer also functions as a Nedd8 ligase and promotes the neddylation of p53 (56). Therefore, MDMX not only can directly interact with p53 and inhibit its activity but also can bind to MDM2 and redirect its ubiquitin ligase activity toward p53 and MDMX itself.

## Amplification and Overexpression of MDMX in Human Cancer and Clinical Outcomes

It has been frequently observed that MDMX is amplified and/or overexpressed in various types of human cancer (as summarized in Table 1). Riemenschneider et al. have firstly reported the aberrant expression of MDMX in malignant gliomas, including glioblastoma (4%) and anaplastic oligodendroglioma (4%) (57). They have found that the malignant gliomas harboring MDMX amplification and overexpression have neither p53 mutation nor MDM2 amplification, supporting the role of MDMX in helping gliomas escape from p53-dependent growth control (57, 58). Similar results have been obtained in other studies (59, 60). However, no association has been found between the aberrant expression of MDMX and the survival of patients with glioblastoma (60). Danovi et al. have further shown that MDMX is overexpressed in breast (19%), lung (18%), and colon (19%) cancer and MDMX amplification (5%) is responsible for MDMX overexpression in breast cancer (61). The amplification and overexpression of MDMX in breast cancer have also been confirmed in Nottingham/Tenovus cohort with a well-characterized series of 990 cases and other studies and have been associated with p53 mutation, breast carcinogenesis, invasive disease, small tumor size, low grade, longer breast carcinoma specific survival (BCSS), and disease-free survival (DFS) (62–65). However, MDMX amplification is rare (1.4%) in colon cancer and its functional significance in this disease still needs further investigations (66). Mancini et al. have found that the expression of full-length *MDMX* (*MDMX-FL*), but not its oncogenic *MDMX-S* variant is significantly associated with the responses of ovarian cancer patients to chemotherapy (67).

**Table 1:** Epidemiological and clinical evidence connecting MDMX and cancer.

**Cancer type**	**Proposed mechanism(s)**	**Clinical outcomes**	**References**
Gliomas	MDMX amplification and overexpression	Helps gliomas escape from p53-dependent growth control; no association with patient survival	(57–60)
Breast cancer	MDMX amplification and overexpression	Associated with carcinogenesis, invasion, small tumor size and low grade, and longer BCSS and DFS	(61–65)
Colon cancer	MDMX overexpression	Promotes carcinogenesis	(61, 66)
Lung cancer	MDMX overexpression	Promotes tumor formation	(61)
Ovarian cancer	NR	Associated with response to chemotherapy	(67)
Gastric cancer	MDMX overexpression	Promotes lymph node metastasis and influences the prognosis	(68, 69)
Bladder cancer	MDMX amplification and overexpression	Associated with high-grade and invasive disease and low recurrence risk; predicts better recurrence-free survival	(70, 71)
Hepatoblastoma	MDMX amplification	NR	(72)
Fibrolamellar hepatocellular carcinoma	MDMX overexpression	NR	(73)
Soft tissue sarcoma	MDMX amplification, MDMX-S overexpression, and high MDMX-S/MDMX-FL ratio	Associated with high tumor grade and poor prognosis	(74, 75)
Osteosarcoma	MDMX amplification, increased MDMX copy number, and high MDMX-S/MDMX-FL ratio	Associated with poor response to chemotherapy, rapid metastatic progression, and poor overall survival	(76–78)
Chronic lymphocytic leukemia	MDMX overexpression	Poor treatment-free survival	(79)
Acute myeloid leukemia	MDMX overexpression	NR	(80)
Mantle cell lymphoma	MDMX overexpression	NR	(81)
Pediatric Burkitt lymphoma	MDMX overexpression	NR	(82)
Salivary gland cancer	MDMX amplification	Associated with high-grade malignancy, lymph node metastasis, advanced tumor size and tumor stage, and poor overall survival	(83)
Papillary thyroid carcinomas	MDMX-S overexpression and downregulation of MDMX mRNA	Associated with multifocality of tumors and advanced tumor stage	(84)
Cutaneous melanoma	MDMX overexpression	Promotes tumorigenesis	(85)
Head and neck squamous carcinomas	MDMX overexpression	NR	(86)
Retinoblastoma	MDMX overexpression	NR	(87)

*BCSS, breast carcinoma specific survival; DFS, Disease-free survival; MDMX-FL, full-length MDMX; MDMX-S, MDMX splice variant; MDMX-S/MDMX-FL ratio, the ratio of the transcript levels of the MDMX-S over MDMX-FL; NR, Not reported*.

The overexpression of MDMX has been found in 36.6% gastric cancer and contributes to lymph node metastasis (68, 69). Although MDMX overexpression can influence the prognosis of gastric cancer, it cannot be used as an independent prognostic factor for this disease (69). The amplification and overexpression of MDMX have also been observed in bladder cancer and has been implicated in high-grade and invasive disease (70). Interestingly, MDMX amplification is significantly associated with low recurrence risk in bladder cancer and predicts better recurrence-free survival of patients with this disease (71). Besides, the amplification and high-level expression of MDMX has been reported in subtypes of liver cancer, i.e., hepatoblastoma and fibrolamellar hepatocellular carcinoma, but the clinical relevance of these findings in liver cancer is not yet clear (72, 73).

MDMX amplification has been reported in both soft tissue sarcomas (17%) and osteosarcomas (35%) (74–77). Bartel et al. have found that MDMX amplification is significantly associated with the poor prognosis of soft tissue sarcomas (74). It has further been found that the MDMX splice variant *MDMX-S* is overexpressed and the ratio of the transcript levels of *MDMX-S* over *MDMX-FL* (the *MDMX-S*/*MDMX-FL* ratio) is increased and correlated with high tumor grade and poor overall survival (74). Lenos et al. have shown an increased copy number of *MDMX* in 71% osteosarcomas, which is correlated to poor response to chemotherapy (78). Further studies have indicated that the high *MDMX-S*/*MDMX-FL* ratio in osteosarcomas is positively associated with the low MDMX protein expression, rapid metastatic progression, and poor overall survival of this disease (78). The overexpression of both *MDMX-FL* and *MDMX-S* has been found in chronic lymphocytic leukemia (CLL) (79), acute myeloid leukemia (80), mantle cell lymphoma (81), and pediatric Burkitt lymphoma (82). Liu et al. have further found a significant increase in the expression of *MDMX-FL* and *MDMX-S* in CLL patients with p53 deletion and/or mutation (79). Importantly, the high expression level of *MDMX-S* mRNA has been associated with the short treatment-free survival (TFS) of CLL patients (79).

Genomic aberration of MDMX has also been observed in salivary gland cancer (SGC) and has been correlated with the poor outcomes of patients with this disease (83). Ach et al. have found that MDMX is amplified in 8.1% salivary gland carcinomas and significantly associated with high-grade malignancy, lymph node metastasis, advanced tumor size and tumor stage, and poor overall survival of SGC patients (83). Interestingly, Prodosmo et al. have reported the *MDMX-S* overexpression and a highly significant downregulation of *MDMX* mRNA in papillary thyroid carcinomas, which has been correlated with the multifocality of tumors and advanced tumor stage (84). In addition, MDMX overexpression has been observed in melanomas (65%) (85), head and neck squamous carcinomas (50%) (86), and retinoblastoma (10%) (87). Several studies on The Cancer Genome Atlas (TCGA) project have reported that SNP rs4245739 in the 3' UTR of the *MDMX* gene is associated with the risk of hormonally mediated cancers (88–91). However, the precise mechanisms and clinical significance should be further investigated.

## MDMX Inhibitors in Preclinical Studies

**Figure 3:**
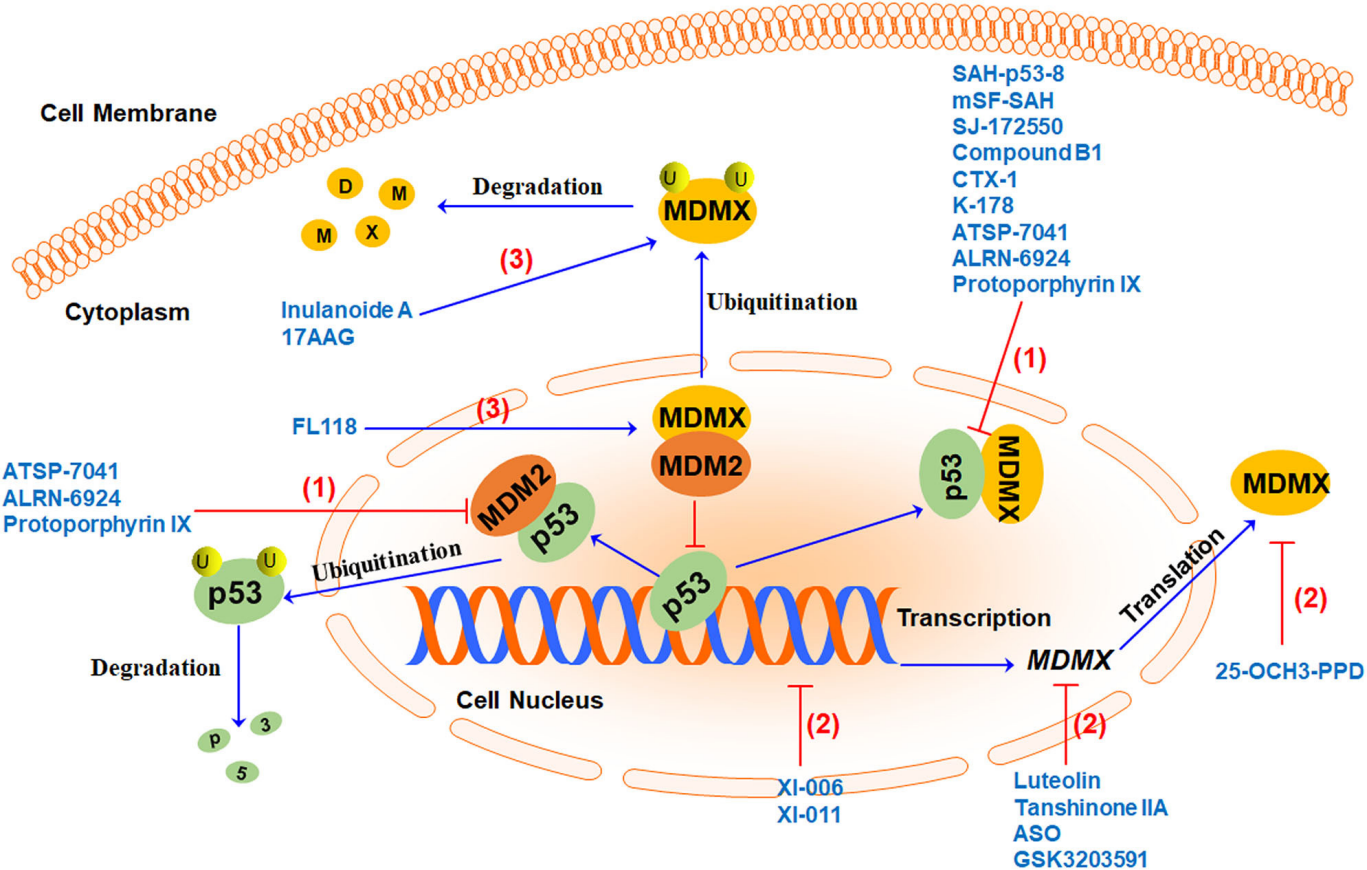
MDMX-targeting strategies and representative MDMX inhibitors. Currently, three MDMX-targeting strategies have been used to develop MDMX inhibitors, including (1) blocking p53-MDMX interaction or p53-MDM2/MDMX interactions to activate p53, (2) inhibiting MDMX expression at multiple levels, and (3) inducing MDMX protein degradation by increasing MDM2-MDMX binding and/or enhancing MDMX ubiquitination.

There is a lot of evidence indicating that cancer often upregulates the expression of MDMX to dampen the tumor-suppressor function of p53. Inhibiting MDMX helps restore the tumor suppressor activity of p53 as well as repress the oncogenic function of MDM2. Therefore, MDMX has recently been demonstrated as a promising therapeutic target for cancer therapy (15). Several MDMX-targeting strategies (Figure 3) have been proposed to develop MDMX inhibitors, including (1) directly blocking p53-MDMX interaction, (2) inhibiting MDMX expression, and (3) inducing MDMX protein degradation. Here we summarize the MDMX inhibitors (Table 2) in recent years and describe their *in vitro* and *in vivo* activities and mechanisms of action.

**Table 2:** MDMX inhibitors and their *in vitro* and *in vivo* activities and mechanisms of action.

**Inhibitors**	**Mechanisms of action**	***In vitro* activity**	***In vivo* activity**	**References**
**STRATEGY 1: BLOCKING MDMX-p53 BINDING**				
SAH-p53-8	Binds to p53-binding pocket of MDMX and blocks the p53-MDMX binding	Inhibits viability and induces apoptosis in cancer cell lines with wild-type p53 and MDMX/MDM2 overexpression	Suppresses tumor growth in JEG-3 xenograft mouse model	(92)
mSF-SAH	Covalently binds to p53-binding pocket of MDMX and blocks the p53-MDMX binding	Nontoxic to p53 *null* Saos-2 cells	NR	(93)
SJ-172550	Covalently but reversibly binds to p53-binding pocket of MDMX, blocks the p53-MDMX binding, and activates p53	Induces apoptosis in retinoblastoma cells with wild-type p53 and MDMX overexpression	NR	(94, 95)
Compound B1	Binds to p53-binding pocket of MDMX and blocks the p53-MDMX binding	NR	NR	(96)
CTX1	Binds to MDMX, blocks the p53-MDMX binding, and activates p53	Induces cancer cell growth arrest and apoptosis alone or in combination with nutlin-3	Extends survival of mice bearing AML xenograft tumors alone or in combination with nutlin-3	(97)
K-178	Inhibits the p53-MDMX binding and activates p53	Inhibits the viability of cancer cell lines with wild-type p53 and MDMX overexpression	Suppresses tumor growth in an HCT116 xenograft mouse model	(98)
ATSP-7041	Binds to MDM2 and MDMX and blocks the p53-MDM2/MDMX bindings	Induces cell cycle arrest in G1 and G2/M phase and apoptosis in cancer cells with wild-type p53	Suppresses tumor growth in the SJSA-1 and MCF-7 xenograft mouse models	(99)
ALRN-6924	Binds to MDM2 and MDMX and blocks the p53-MDM2/MDMX bindings	Inhibits cell proliferation and clonogenic capacity and induces cell cycle arrest and apoptosis in AML cells with wild-type p53	Exerts robust anti-leukemic efficacy in AML xenograft mouse models	(100)
Protoporphyrin IX	Blocks the p53-MDM2/MDMX bindings and stabilizes p53 and TAp73	Inhibits proliferation and induces apoptosis in CLL cells without affecting normal cells	NR	(101)
**STRATEGY 2: INHIBITING MDMX EXPRESSION**				
XI-006 (NSC207895)	Inhibits *MDMX* promoter activity	Induces cancer cell growth arrest and apoptosis and enhances the anticancer activity of nutlin-3	NR	(102, 103)
XI-011 (NSC146109)	Inhibits *MDMX* promoter activity	Induces breast cancer cell apoptosis	NR	(104)
Luteolin	Decreases MDMX expression by up-regulating microRNA-34a-5p	Inhibits cancer cell proliferation and induces apoptosis	Suppresses tumor growth in an H460 xenograft mouse model	(105)
Tanshinone IIA	Inhibits *MDMX* mRNA synthesis	Induces cancer cell apoptosis alone or in combination with doxorubicin	NR	(106)
25-OCH3-PPD	Inhibits MDMX expression and MDM2-MDMX interaction and induces MDM2 degradation	Inhibits prostate cancer cell proliferation, migration, and invasion	Suppresses prostate tumorigenesis and metastasis in the TRAMP mouse model	(107)
ASO	Decreases expression level of *MDMX-FL*	Reduces the viability of the melanoma and DLBCL cells	Suppresses tumor growth in PDX models of melanoma and DLBCL	(108)
GSK3203591	Induces alternative splicing of *MDMX* and activates p53 by inhibiting PRMT5	Inhibits cancer cell growth and survival	Suppresses tumor growth in lymphoma xenograft mouse models	(109)
**STRATEGY 3: INDUCING MDMX PROTEIN DEGRADATION**				
FL118	Enhances MDM2-mediated MDMX ubiquitination and degradation	Inhibits cell growth and induces p53-dependent senescence and p53-independent apoptosis in colorectal cancer cells	NR	(110)
Inulanolide A	Binds to the RING domains of MDM2 and MDMX and induces their ubiquitination and degradation	Inhibits cancer cell growth, proliferation, migration, and invasion and induces G2/M phase arrest and apoptosis	Suppresses tumor growth and lung metastasis in the MDA-MB-231 orthotopic mouse model	(111, 112)
17AAG	Induces robust MDMX degradation and activates p53	Induces cancer cell apoptosis alone or in combination with nutlin-3	Suppresses tumor growth in a RKO xenograft mouse model alone or in combination with nutlin-3	(113)

*AML, Acute myeloid leukemia; ASO, Antisense oligonucleotide; DLBCL, Diffuse large B cell lymphoma; CLL, Chronic lymphocytic leukemia; NR, Not reported*.

### Blocking p53-MDMX Interaction

Due to advances in understanding the structure and binding mechanisms of p53-MDM2 and p53-MDMX complexes, many inhibitors have been developed to dually inhibit the interactions of p53-MDM2/MDMX, which have been comprehensively discussed in the recent review articles (26–29). Several specific p53-MDMX binding inhibitors, including, but not limited to, SAH-p53-8 (92), SJ-172550 (94), compound B1 (96), CTX1 (97), and K-178 (98) have recently been discovered and shown promising anticancer efficacy and safety profiles in preclinical models *in vitro* and *in vivo* (Table 2). Bernal et al. have designed and synthesized a stapled p53 helix, named SAH-p53-8 that specifically binds to MDMX with strong affinity (*K*_D_ = 2.3 nM) (92). SAH-p53-8 has further been shown to block the p53-MDMX binding and activate the p53 signaling pathway, thus inhibiting cancer cell viability, inducing apoptosis, and overcoming MDMX-mediated cancer chemoresistance *in vitro* and *in vivo*. Hoppmann and Wang have designed and synthesized covalent peptide inhibitors of p53-MDMX binding based on the structure of SAH-p53-8, leading to the discovery of mSF-SAH (93). Although mSF-SAH has shown more efficient inhibition on the p53-MDMX interaction than SAH-p53-8, the anticancer efficacy of this new peptide is yet to be determined.

Reed et al. have performed a high-throughput screening of the St. Jude chemical library (containing 295,848 unique compounds) by using a fluorescence polarization (FP)-based MDMX-p53 binding assay as well as a cell-based assay and identified 11 representative compounds (94). Among these 11 compounds, SJ-134433 and SJ-044557 have been found to covalently modify MDMX and thus be abandoned for further investigations, whereas SJ-172550 has been shown to reversibly bind to the p53-binding pocket of MDMX, block the p53-MDMX binding, activate wild-type p53, and induce apoptosis in retinoblastoma cells with MDMX overexpression (94). However, the same group has also found that SJ-172550 covalently but reversibly interacts with MDMX and causes a conformational change of this protein, which consequently loses the ability to bind p53 (95). This complicated binding mechanism of SJ-172550 hinders its further evaluation as an MDMX inhibitor (95). Boltjes et al. have recently generated a peptidomimetic MDMX inhibitor, named compound B1 and examined its inhibitory effects on MDMX-p53 binding (*K*_i_ = 5.5 μM) using the FP-based assay. However, the anticancer efficacy of compound B1 has yet to be determined (96).

Karan et al. have also performed a cell-based screening of a compound library (over 20,000 small molecules) and identified a lead compound CTX1, which induces strong p53-dependent activation in MCF-7 cells expressing a high level of MDMX (97). Further studies have shown that CTX1 directly binds to MDMX (*K*_D_ = 450 nM), prevents the formation of p53-MDMX complex, and activates p53 without causing DNA damage (97). CTX1 alone or in combination with an MDM2 inhibitor nutlin-3 has been found to induce cancer cell growth arrest and apoptosis *in vitro* and extend the survival of mice bearing acute myeloid leukemia (AML) xenograft tumors *in vivo* (97). In addition, Uesato et al. have designed and synthesized a class of o-aminothiophenol derivatives as selective p53-MDMX binding inhibitors. The lead compound K-178 has been shown to activate the p53 signaling and inhibit the viability of cancer cell lines with wild-type p53 and overexpressing MDMX, including human breast (MCF-7), lung (A427), and colon (HCT116) cancer cell lines. It has also been demonstrated that K-178 suppresses tumor growth in an HCT116 xenograft mouse model without affecting the average mouse body weight during the treatment period (98). Further studies on both CTX1 and K-178 in more clinically relevant cancer models are needed for the further development of these MDMX inhibitors as anticancer drugs.

Several dual inhibitors of the p53-MDM2/MDMX interactions have been identified and shown promising efficacy *in vitro* and *in vivo*. Optimization of p53 stapled peptides has been considered as a reasonable strategy to discover effective p53 activators, which leads to the identification of ATSP-7041 and ALRN-6924 (99, 100). Chang et al. have reported that ATSP-7041 directly binds to both MDM2 and MDMX with high affinities (*K*_i_ = 0.9 and 7 nM, respectively) and effectively disrupts the p53-MDM2/MDMX bindings, thereby activating p53 signaling in p53 wild-type cancer cells. ATSP-7041 has been found to induce cell cycle arrest in G1 and G2/M phase and apoptosis *in vitro* and suppress xenograft tumor growth *in vivo* in a p53-dependent manner (99). Importantly, ATSP-7041 has shown favorable DMPK and tissue distribution properties, which are critical for the further development of this peptide as an anticancer drug. Moreover, Carvajal et al. have identified a stapled α-helical peptide ALRN-6924 as a dual p53-MDM2/MDMX inhibitor, which has recently entered phase I clinical trial (100). ALRN-6924 has also shown nanomolar binding affinities to both MDM2 and MDMX (*K*_i_ = 1.3 and 7 nM, respectively). It has further been found that ALRN-6924 inhibits the p53-MDM2/MDMX interactions and activates p53, thus inhibiting cell proliferation and clonogenic capacity and causing cell cycle arrest and apoptosis in AML cells harboring wild-type p53 (100). ALRN-6924 has also shown robust anti-leukemic efficacy in primary patient-derived AML cells and AML xenograft mouse models. Besides, protoporphyrin IX (PpIX), a metabolite of the clinically approved drug aminolevulinic acid has recently been characterized as a dual inhibitor of the p53-MDM2/MDMX interactions (101). Jiang et al. have found that PpIX inhibits cell proliferation and promotes apoptosis in B-cell chronic lymphocytic leukemia cells without significant effects on normal cells. The PpIX-induced apoptosis in leukemia cells has been attributed to the stabilization of p53 and TAp73 proteins by the compound (101).

### Inhibiting MDMX Expression

Direct inhibition of MDMX expression has been demonstrated as an effective approach for p53 activation. Several small-molecule inhibitors have recently been discovered to down-regulate MDMX expression and up-regulate the expression of p53 and its target genes, e.g., p21. Wang et al. have carried out a promoter-based screening of NCI Diversity-Set chemical library and identified a lead compound, XI-006 (NSC207895), which decreases both the MDMX mRNA and protein levels through repressing *MDMX* promoter activity (102). Further studies have found that XI-006 activates p53 and increases the expression of p53 target genes related to cell cycle arrest (e.g., p21) and apoptosis (e.g., PUMA, BAX, and PIG3), causing the accumulation of MCF-7 cells at subG0/G1 phase and apoptosis (102). It has also been shown that XI-006 additively enhances nutlin-3-induced p53 activation and its inhibitory effects on cancer cell viability *in vitro* (102). Pishas et al. have further demonstrated the anticancer activity of XI-006 in Ewing and osteosarcoma cell lines *in vitro* (103). However, neither the protein/mRNA levels of MDMX nor the p53 status has played an essential role in XI-006-mediated apoptosis in these cell lines, suggesting a complicated mechanism of action (103). In addition, Wang and Yan have reported another MDMX inhibitor, termed XI-011 (NSC146109), which induces apoptosis in breast cancer MCF7 cells by activating p53 (104). Further studies have shown that XI-011 decreases *MDMX* mRNA level by repressing *MDMX* promoter activity and MDMX expression plays an important role in the anticancer activity of XI-011 (104). Further investigations on the precise mechanisms of action for both XI-006 and XI-011 are warranted.

Jiang et al. have reported that luteolin exerts its anticancer activity by enhancing microRNA-34a-5p-mediated MDMX repression (105). It has been found that luteolin inhibits cell proliferation and induces apoptosis in non-small cell lung cancer (NSCLC) A549 and H460 cell lines *in vitro* and suppresses tumor growth in an H460 xenograft mouse model *in vivo*. The molecular mechanism studies have shown that luteolin up-regulates the expression of miR-34a-5p in NSCLC cells, which consequently leads to the down-regulation of its downstream target MDMX as well as the upregulation of p53 and p21 (105). The importance of the miR-34a-5p-MDMX axis in the anticancer activity of luteolin has also been validated using the transient transfection experiments of a miR-34a-5p inhibitor (105). Zu et al. have recently characterized tanshinone IIA as a new MDMX inhibitor (106). Tanshinone IIA has been found to inhibit MDMX expression by repressing *MDMX* mRNA synthesis, which leads to the downregulation of IAP3 (Inhibitor of apoptosis 3) (106). Further studies have shown that tanshinone IIA not only induces the apoptosis of H1299 cells but also enhances the doxorubicin-induced apoptosis (106).

Ginsenosides are a class of natural products with diverse biological activities (114). Wang et al. have identified a novel ginsenoside, 25-OCH3-PPD that possesses potent anticancer activities in preclinical cancer models *in vitro* and *in vivo*, with minimal host toxicity (115, 116). A recent study by the same group has reported that 25-OCH3-PPD inhibits MDMX expression and MDMX-MDM2 binding, thereby inducing the degradation of MDM2 (107). They have found that 25-OCH3-PPD inhibits the viability, proliferation, migration, and invasion of prostate cancer LNCaP, PC3, and DU145 cells *in vitro*, regardless of the p53 and androgen receptor statuses. Further *in vivo* studies have shown that 25-OCH3-PPD prevents prostate tumorigenesis and metastasis in the TRAMP mouse model without causing any significant host toxicity (107). However, the detailed mechanisms for the inhibitory effects of 25-OCH3-PPD on MDMX expression should be further investigated.

Antisense oligonucleotides (ASOs) have been used to efficiently modulate *MDMX* mRNA splicing (108). Dewaele et al. have demonstrated that enhanced exon 6 inclusion increases *MDMX-FL* expression level in human melanoma while exon 6 skipping causes *MDMX-S* production and decreases the expression level of *MDMX-FL*. It has further been shown that ASO-mediated exon 6 skipping decreases MDMX protein level and inhibits the growth of melanoma and diffuse large B cell lymphoma *in vitro* and *in vivo*. Because MDMX plays an important role in cancer cell chemoresistance, it is not surprising that the MDMX inhibition by ASO-mediated exon 6 skipping enhances the sensitivity of melanoma cells to BRAFV600E inhibitors *in vitro* and *in vivo* (108).

Protein arginine methyltransferase 5 (PRMT5) has recently been identified as a master regulator of splicing, which regulates MDMX abundance by controlling its alternative splicing (117). Herein, Gerhart et al. have examined the effects of PRMT5 inhibitors on the p53-MDMX axis and their anticancer efficacy (109). It has been found that PRMT5 inhibition by a specific inhibitor GSK3203591 induces the alternative splicing of *MDMX* and activates the p53 pathway. Further studies have shown that GSK3203591 inhibits cancer cell growth and survival *in vitro* and suppresses tumor growth in lymphoma xenograft mouse models *in vivo* (109).

### Inducing MDMX Protein Degradation

Given that the formation of MDM2-MDMX complex stabilizes MDM2 protein, enhances its E3 ubiquitin ligase activity, and promotes the ubiquitination and degradation of MDMX, the MDM2-MDMX binding has been considered as a promising target for cancer therapy. Ling et al. have reported that FL118, a previously discovered inhibitor of survivin expression inhibits p53 ubiquitination but promotes MDMX protein degradation (110). They have further found that FL118 moderately increases the MDM2-MDMX binding and enhances MDM2-mediated MDMX ubiquitination and degradation. Importantly, MDMX overexpression has also increased FL118-induced death of HCT116 cancer cells, confirming the importance of MDMX expression for its anticancer activity. Moreover, FL118 has been shown to induce p53/p21-dependent senescence and p53-independent apoptosis in HCT116 cells (110).

Sesquiterpenoids are a class of structurally diverse and pharmacologically active natural products (118, 119). We have previously identified several dimeric sesquiterpenoids with potent anticancer activity in preclinical breast cancer models *in vitro* and *in vivo* (120, 121). Among these dimeric sesquiterpenoids, Inulanolide A has recently been shown to directly bind to the RING domains of MDM2 and MDMX, disrupt the MDM2-MDMX interaction, and promote the ubiquitination and proteasomal degradation of both proteins (112). We have shown that Inulanolide A inhibits cell viability and proliferation, induces G2/M-phase cell cycle arrest and apoptosis, and prevents migration and invasion in breast and prostate cancer cell lines *in vitro* (111, 112). Its *in vivo* inhibitory effects on tumor growth and metastasis have also been demonstrated in an MDA-MB-231 orthotopic mouse model (111). However, further evaluation of Inulanolide A in more clinically relevant models is warranted for the development of this compound as an anticancer agent.

Vaseva et al. have recently found that the Hsp90 inhibitor 17-allylamino-17-demethoxygeldanamycin (17AAG) induces cancer cell apoptosis and synergistically enhances nutlin-3-induced apoptosis in a p53-dependent manner (113). 17AAG has also been shown to suppress tumor growth in an H460 xenograft mouse model alone or in combination with nutlin-3. Mechanistically, 17AAG has been found to induce MDMX protein degradation, thereby disrupting the p53-MDMX interaction and activating p53 (113). However, the molecular mechanisms for 17AAG-induced MDMX degradation remain to be determined in the future.

## Conclusion and Future Directions

Over the last two-and-a-half decades, MDMX has been developed from the status of the little brother of its well-studied and important “older” relative MDM2 to another key negative regulator of p53 in embryonic and, perhaps most importantly, cancer cells. Given that MDMX is frequently amplified and/or overexpressed in various types of human cancer (as summarized in Table 1) and plays an essential role in controlling the p53-MDM2/MDMX loop, MDMX has been demonstrated as a promising molecular target for cancer therapy. More and more researchers invest in the discovery of natural-product and rationally designed MDMX inhibitors and demonstrate their anticancer efficacy and mechanism(s) of action in preclinical cancer models *in vitro* and *in vivo*. Their main focus has been on the design and development of p53-MDMX binding inhibitors by targeting the p53-binding pocket of MDMX, such as SAH-p53-8 (92), mSF-SAH (93), SJ-172550 (94), compound B1 (96), CTX1 (97), and K-178 (98). All these inhibitors exert their anticancer activity through activating wild-type p53 and the transcription of its downstream target genes related to cell cycle arrest (e.g., p21) and apoptosis (e.g., Puma and Bax). However, the mutations and deletions of p53 are common genetic events in human cancer and lead to the limited efficacy of the p53-MDMX binding inhibitors in such types of cancer (20, 122).

Several small-molecule inhibitors of MDMX expression have been developed to inhibit both the p53-dependent and the p53-independent functions of MDMX, such as XI-006 (NSC207895) (102), XI-011 (104), luteolin (105), tanshinone IIA (106), 25-OCH3-PPD (107), ASO (108), and GSK3203591 (109). However, inhibiting the expression of a molecular target often causes unexpected adverse effects (123, 124). Of note, the regulatory mechanisms driving MDMX expression have not yet been fully elucidated, which is critical for developing such type of MDMX inhibitors. Recent studies have been performed to identify small-molecule inducers of MDMX protein degradation, such as FL118 (110), Inulanolide A (111), and 17AAG (113). Although targeting MDM2-MDMX interaction to enhance MDM2-mediated MDMX ubiquitination is an effective approach for promoting MDMX degradation, other potential strategies are also valuable and need to be examined. For example, inhibiting USP2a (ubiquitin-specific protease 2a)-mediated MDMX deubiquitination and stabilization and developing MDMX PROTACs (proteolysis targeting chimeras) should be considered for developing selective MDMX inhibitors for cancer therapy (125, 126).

The recently identified alternative splicing-dependent mechanism for MDMX overexpression in cancer has offered an alternative option for inhibiting MDMX (15, 127, 128). The highly expressed MDMX further inhibits p53 activation and compromises the p53-dependent cellular response to ionizing radiation and ribosomal stress as well as to the treatment of MDMX inhibitors (129, 130). The ectopically expressed MDMX-S protein, an MDMX spliceosome has shown a higher binding affinity to p53 and thus causes more potent inhibition on p53 than full-length MDMX. Although the expression of *MDMX-S* mRNA is often observed, the endogenous MDMX-S protein is undetectable in any normal or cancer cell lines, which implies that the endogenous MDMX-S may be a very unstable protein and the switch from MDMX-FL to MDMX-S may play a critical role in controlling the protein level of MDMX-FL and activating p53 in normal and/or cancer cells (128, 131). Besides, MDMX-211, another aberrantly spliced form of MDMX spliceosome has been found to bind to and stabilize MDM2 protein (132). Although there is no direct binding of MDMX-211 to p53, MDMX-211 also stabilizes p53 by counteracting MDM2-mediated p53 degradation (132). These MDMX spliceosomes may also be targeted for developing anticancer drugs due to their pivotal roles in regulating the protein stabilities and activities of MDMX, MDM2, and p53.

Paradoxically, increasing evidence has suggested a tumor suppressor role for MDMX under different stress conditions. In response to DNA damage, MDMX has been found to stably localize at the mitochondria, in which it enhances p53 phosphorylation at serine 46 (Ser46) and its mitochondrial localization, facilitates the p53Ser46^p^-Bcl2 (B-cell lymphoma 2) binding, and p53-HIPK2 (Homeodomain-interacting protein kinase 2) functional interaction, and promotes p53-mediated apoptosis (67, 125, 133). Besides, DNA damage and other stress conditions activate c-Abl tyrosine kinase that phosphorylates MDMX at tyrosine 99 (Tyr99), thereby reducing the MDMX-p53 binding and activating p53-dependent apoptosis pathway (134). It has also been shown that MDMX enhances the sensitivity of cancer cells to cisplatin (67, 135, 136). Although the pro-apoptotic roles of MDMX in cancer remain to be determined, it is worthy to be investigated whether MDMX inhibitors attenuate the anticancer efficacy of platinum-based chemotherapy, especially cisplatin. Considering the critical role of MDMX expression in cisplatin-induced cancer cell death, the MDMX inhibitor and cisplatin combination may exert an antagonistic effect and even cause chemoresistance. More detailed investigations are warranted to answer these questions.

In conclusion, the accumulating knowledge about the roles of MDMX in the p53-MDM2/MDMX loop, its p53-independent oncogenic functions, and the regulatory mechanisms for MDMX expression and protein stability may provide new opportunities to develop novel MDMX inhibitors. A better understanding of these MDMX inhibitors, especially their selectivity, efficacy, pharmacological, and toxicological properties is critical for moving them forward from bench to clinical trials.

## Author Contributions

J-JQ conceptualized the manuscript. D-HY, Z-YX, SM, LY, X-DC, and J-JQ collected the literature, wrote the manuscript, and made the figures. J-JQ edited and made significant revisions to the manuscript. All authors read and approved the final manuscript.

## Conflict of Interest

The authors declare that the research was conducted in the absence of any commercial or financial relationships that could be construed as a potential conflict of interest.
